# Reconstructed Order Analysis-Based Vibration Monitoring under Variable Rotation Speed by Using Multiple Blade Tip-Timing Sensors

**DOI:** 10.3390/s18103235

**Published:** 2018-09-26

**Authors:** Zhongsheng Chen, Jianhua Liu, Chi Zhan, Jing He, Weimin Wang

**Affiliations:** 1 College of Electrical & Information Engineering, Hunan University of Technology, Zhuzhou 412007, China; hejing@163.com; 2 Science and Technology on Integrated Logistics Support Laboratory, National University of Defense Technology, Changsha 410073, China; chizhan_nudt@163.com; 3 College of Traffic Engineering, Hunan University of Technology, Zhuzhou 412007, China; 4 Beijing Key Laboratory of Health Monitoring and Self-recovery for High-end Mechanical Equipment, Beijing University of Chemical Technology, Beijing 100029, China; wwm@mail.buct.edu.cn

**Keywords:** vibration monitoring, multiple blade tip-timing sensors, variable rotation speeds, angular sampling, reconstructed order analysis

## Abstract

On-line vibration monitoring is significant for high-speed rotating blades, and blade tip-timing (BTT) is generally regarded as a promising solution. BTT methods must assume that rotating speeds are constant. This assumption is impractical, and blade damages are always formed and accumulated during variable operational conditions. Thus, how to carry out BTT vibration monitoring under variable rotation speed (VRS) is a big challenge. Angular sampling-based order analyses have been widely used for vibration signals in rotating machinery with variable speeds. However, BTT vibration signals are well under-sampled, and Shannon’s sampling theorem is not satisfied so that existing order analysis methods will not work well. To overcome this problem, a reconstructed order analysis-based BTT vibration monitoring method is proposed in this paper. First, the effects of VRS on BTT vibration monitoring are analyzed, and the basic structure of angular sampling-based BTT vibration monitoring under VRS is presented. Then a band-pass sampling-based engine order (EO) reconstruction algorithm is proposed for uniform BTT sensor configuration so that few BTT sensors can be used to extract high EOs. In addition, a periodically non-uniform sampling-based EO reconstruction algorithm is proposed for non-uniform BTT sensor configuration. Next, numerical simulations are done to validate the two reconstruction algorithms. In the end, an experimental set-up is built. Both uniform and non-uniform BTT vibration signals are collected, and reconstructed order analysis are carried out. Simulation and experimental results testify that the proposed algorithms can accurately capture characteristic high EOs of synchronous and asynchronous vibrations under VRS by using few BTT sensors. The significance of this paper is to overcome the limitation of conventional BTT methods of dealing with variable blade rotating speeds.

## 1. Introduction

High-speed blades are key mechanical rotating components in turbo-machinery, such as engine compressor and turbine blades. High cycle fatigue due to low stress and high-frequency vibrations cause different kinds of blade damage or even catastrophic faults. Statistical data have shown that over 60% of the overall engine faults are caused by vibrations. Furthermore, blade faults have accounted for more than 70% of the overall vibration-induced faults [1]. Thus, on-line vibration monitoring of high-speed blades is urgent from the viewpoints of safety, reliability, availability, and maintenance [2]. As blades rotate continuously during operating, it is difficult to carry out on-line vibration monitoring. Currently, there are two classes of methods, namely contact and non-contact monitoring. For contact vibration monitoring, strain gauges are mounted on a blade surface, and signals are sampled by using a slip-ring [3]. This class of method has several intrinsic drawbacks. First, we need to use at least a strain gauge to monitor each blade. This results in numerous strain gauges being used for monitoring the whole engine. Strain gauges themselves may affect the vibration characteristics of a blade. Second, the lifespan of a strain gauge is limited due to the challenging operational environments, so they need to be replaced regularly, and the cost is high. Third, a high-speed slip ring is needed to transmit signals of all strain gauges, the expense of which is also very high. To overcome these shortcomings, non-contact blade vibration monitoring methods have arisen in recent years, such as laser Doppler vibrometry (LDV) [4]. Stationary LDV measurement can be used to detect all the blades as they pass in front of the sensor through non-harmonic Fourier analysis [5,6,7]. Recently, blade tip-timing (BTT) has become another promising non-contact measurement approach [8,9]. BTT uses the times at which the blade tips pass the casing-mounted probes to obtain all-blade vibrations simultaneously.

A review of BTT vibration analysis methods can be found in References [10,11]. To date, a few methods have been proposed to identify synchronous or asynchronous blade vibrations at constant rotation speed [12,13]. Lawson and Ivey proposed measuring blade vibration amplitude through dual capacitance probe tip timing [14]. Bastami et al. used two BTT sensors to identify the frequency and amplitude of asynchronous blade vibration by using the least mean square algorithm [15]. Dimitriadis et al. studied the effects of the various dynamic phenomena during synchronous vibrations on the BTT technique itself and pointed out that there is no single BTT data analysis method to address all these phenomena [16]. Battiato et al. evaluated the accuracy of the tip-timing system by measuring the forced response of rotating bladed disks under synchronous excitations [17]. Diamond et al. compared three blade tip timing algorithms for estimating synchronous turbomachine blade vibration [18]. However, under-sampling is an intrinsic drawback of BTT methods, so frequency aliasing always exists in the direct spectral analysis. To overcome it, Chen et al. proposed under-sampled blade vibration reconstruction methods by using the Shannon sampling theorem and wavelet packet transform [19,20]. Hu et al. presented a novel reconstruction method for non-uniformly under-sampled BTT data based on the periodically non-uniform sampling theorem [21]. Lin et al. reconstructed unknown multi-mode blade vibration signals based on sparse representation and compressed sensing [22].

In practice, the rotating speed is impossible to keep constant due to unstable airflow, load instability, and other dynamic factors. Furthermore, blade damage is constantly formed and accumulated during variable operational conditions due to their frequently passing resonant regions. In this sense, variable rotation speed (VRS) may have positive effects on blade vibration monitoring. For example, VRS may result in nonlinear vibration phenomenon [23], such as amplitude modulation, frequency modulation, and phase modulation. In this case, it may be easier to detect incipient blade damage. Therefore, it is important to perform vibration monitoring under VRS. However, existing methods result in significant estimation errors due to VRS. BTT sensors are only mounted around the circumference of the bladed disk, so BTT sampling can be looked at as an angular sampling process. As we know, angular sampling-based order analysis has been widely used for vibration signals in rotating machinery with variable speeds [24,25,26,27,28,29,30,31]. Fyfe et al. [29] discussed the effects of various factors on its accuracy in the computed order tracking method. An extended work was discussed by Bosssley et al. [30] which focused on the assessment of the accuracy of three different order tracking methods. As a BTT system is only a hardware-implemented order analysis configuration, the computed order tracking method will not be used. The angular sampling rate of the BTT method depends completely on the number of sensors so that the sampling order of blade vibration signals is always much less than engine orders (EOs) of interest. In this case, blade vibration signals in the angular domain are well under-sampled, so that existing order analysis and reconstruction methods will not work. To our knowledge, no related works have been done to reconstruct true vibration characteristics from under-sampled BTT vibration signals under VRS.

This paper aims to explore a reconstructed order analysis-based BTT vibration monitoring method under VRS. Moreover, both uniform and non-uniform BTT sensor configuration are considered. First, BTT vibration signals in the domain are transformed into angularly sampled signals. Then, angular-domain BTT vibration reconstruction algorithms under VRS are proposed for both uniform and non-uniform BTT sensor configurations, respectively. Based on them, order analysis is used to extract high EOs of synchronous and asynchronous vibrations. The remainder of the paper is organized as follows: In Section 2, basic problems of BTT vibration reconstruction under VRS are described, and the basic structure of angular sampling-based BTT vibration monitoring under VRS is proposed. Next BTT vibration reconstruction algorithms under VRS are presented in Section 3. In Section 4, numerical simulations are done. Then a test rig is built, and experiments are carried out to validate the proposed method in Section 5. Finally, conclusions are summarized in Section 6.

## 2. Angular Sampling-Based BTT Vibration Monitoring under Variable Rotation Speed

### 2.1. Basic Principles of BTT-Based Vibration Monitoring

**Figure 1:**
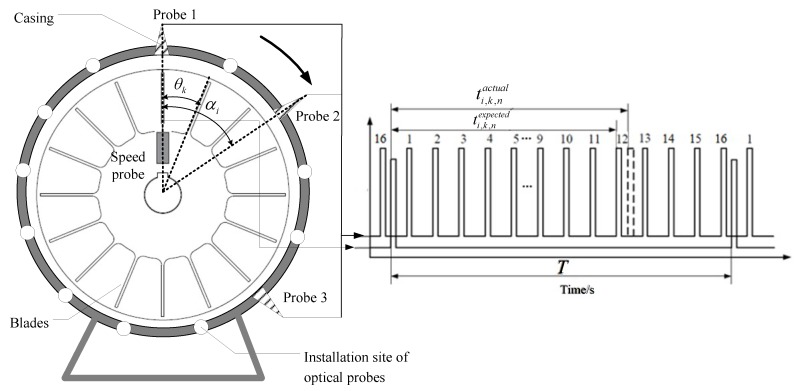
Basic principle of the blade tip-timing (BTT) method.

The basic principle of a BTT monitoring system is shown in Figure 1. First, I BTT sensors are embedded into a stationary casing around a bladed-disk with K blades. In practice, BTT sensors can be optic fiber, eddy current, microwave or capacitive sensors. At the same time, a once-per-revolution sensor is mounted in front of the rotating shaft as a reference sensor. The angular positions of the ith(1≤i≤I) BTT sensor and the kth(1≤k≤K) blade are denoted as $\alpha_{i}$ and $\theta_{k}$, respectively.

The basic idea of the BTT method is to measure the arrival times as each blade passes each BTT sensor. When there are no blade vibrations under ideal conditions, the passage times of each blade will only be a function of rotating speed, rotating radius, and its circumferential position. Once these parameters are fixed, the blade passage times are also deterministic. However, when blade vibrations happen, the blades will pass BTT sensors earlier or later than normal intervals. Thus, the blade passing times will deviate from those under undisturbed conditions and a time difference series will be generated for each blade. Then vibration displacements of each blade can be calculated based on these time differences. The BTT method can be used to measure all-blade vibrational displacements, which is superior to other contact monitoring methods. The details are summarized as follows:

Let the bladed-disk rotate clockwise at a constant speed. Under ideal conditions, the expected arrival times of the *k*th blade passing the *i*th BTT sensor can be calculated as:

(1) $$t_{i,k,n}^{expected}=\frac{1}{2\pi f_{r}}\left[2\pi \left(n-1\right)+\alpha_{i}-\theta_{k}\right],n=1,2,\ldots ,N\;$$

where $f_{r}$ is the rotating frequency and n denotes the nth revolution.

The actual arrival times of the *k*th blade passing the *i*th BTT sensor are measured as $t_{i,k,n}^{actual}$. Then the time difference series is calculated as:

(2) $$\Delta t_{i,k,n}=t_{i,k,n}^{expected}-t_{i,k,n}^{actual}\;$$

Furthermore, vibration displacements of the *k*th blade measured by the *i*th BTT sensor can be calculated as:

(3) $$d_{i,k}[n]=2\pi f_{r}R\Delta t_{i,k,n}=2\pi f_{r}R\left(t_{i,k,n}^{expected}-t_{i,k,n}^{actual}\right)\;$$

where R is the rotating radius of the blade tip.

It can be seen that I vibration displacements of each blade are measured during each revolution due to I BTT sensors. Thus, the BTT sampling frequency $f_{BTT}$ of each blade can be defined as $I\times f_{r}$ if the BTT sensors are mounted uniformly. In engineering applications, the number of BTT sensors I is often small due to the restrictions of spaces and costs, so the sampling frequency $f_{BTT}$ is always less than the natural frequencies of a blade. Thus, BTT vibration signals are typically under-sampled. In this case, we cannot directly use sampled vibration displacements to perform vibration monitoring, and we need to reconstruct the vibration signal to obtain true vibration characteristics of a blade.

### 2.2. Problem Statements of BTT Vibration Reconstruction under VRS

By now, several works have been done to reconstruct under-sampled BTT vibration signals [17,18,19], which are mainly based on Equations (1)–(3). According to Equations (1) and (3), the rotating frequency $f_{r}$ is assumed to be a constant in previous works. In practice, however, this assumption is rarely satisfied due to many factors, such as variable operation conditions, unstable airflows, rotor imbalances, and so on. In this case, estimated vibration displacements are not accurate, which will cause existing reconstruction methods to be unusable.

When considering VRS, the rotating frequency $f_{r}$ should not be a constant. In this case, the period of the *i*th revolution is denoted as $T_{i}$. The expected arrival times of the *k*th blade passing the *i*th BTT sensor can be calculated as:

(4) $${\tilde{t}}_{i,k,n}^{expected}=\sum_{i=1}^{n-1}T_{i}+\frac{\alpha_{i}-\theta_{k}}{2\pi }T_{n},n=1,\ldots ,N\;$$

Furthermore, when the blade has a vibration displacement $d\left(t_{i,k,n}^{}\right)$ at that time, the actual arrival times can be expressed as:

(5) $${\tilde{t}}_{i,k,n}^{actual}=\sum_{i=1}^{n-1}T_{i}+\frac{\alpha_{i}-\theta_{k}}{2\pi }T_{n}-\frac{d\left(t_{i,k,n}^{}\right)}{2\pi R}T_{n}\;$$

Then blade vibration displacements under VRS can be estimated as:

(6) $$\begin{array}{ll} {\tilde{d}}_{i,k}\left[n\right] & =2\pi R\left({\tilde{t}}_{i,k,n}^{expected}-{\tilde{t}}_{i,k,n}^{actual}\right)/T_{n}\\ & =2\pi R\left(\sum_{i=1}^{n-1}T_{i}+\frac{\alpha_{i}-\theta_{k}}{2\pi }T_{n}-{\tilde{t}}_{i,k,n}^{actual}\right)/T_{n} \end{array}$$

In practice, ${\tilde{t}}_{i,k,n}^{actual}$ can be measured by the BTT monitoring system. By comparing Equation (6) with Equation (3), we can see that blade vibration displacements under VRS are significantly different from those under constant speeds. In particular, it brings two main obstacles to existing reconstruction methods. The first is that the rotating period is not fixed so that it is difficult to calculate $f_{r}$ accurately in Equation (3). The second is that the BTT sampling process is non-uniform, even though BTT sensors are mounted uniformly around the casing. In this case, previous methods cannot be used [19,20,21,22]. Therefore, new reconstruction algorithms should be explored for BTT vibration monitoring under VRS to the best of our knowledge this has not been reported before.

### 2.3. Basic Structure of Angular Sampling-Based BTT Vibration Monitoring under VRS

As we know, high cycle fatigue (HCF) is a common failure mode of high-speed rotating blades. Generally speaking, blade vibrations are a major reason for generating HCFs, which can be classified into synchronous and asynchronous vibrations. Frequencies of unsteady aerodynamic forces due to aerodynamic instabilities, such as wakes of upstream blades, struts, and burners, are usually integer multiples (called engine order) of the rotation speed of the blade. Then, vibrations of the blade which occur at the engine order (EO) are termed as synchronous vibrations. Whereas, some flow instabilities appear at a distinct frequency which is not an integer multiple of the rotor speed so that it is not synchronous with the rotating speed. In addition, flow instability naturally occurs at one frequency locking on one of the nearby natural frequencies of the blade, leading to shifting, somewhat, the frequency of the response. Such vibration is termed as asynchronous vibration. In practice, synchronous vibrations enlarge vibration amplitudes of rotating blades, so they are more dangerous to rotating blades than asynchronous vibrations. On the other hand, blade damage also causes asynchronous vibrations. Thus, BTT vibration monitoring aims to accurately identify both these characteristic vibrations.

As for the under-sampling problem of BTT techniques, angular re-sampling in vibration monitoring of rotating machinery is introduced in this paper, and an idea of angular sampling-based BTT vibration monitoring is proposed. Its basic principles are as follows: The I BTT sensors are assumed to be uniformly mounted around the casing, so that angle between any two adjacent blades is the same. In this case, blade vibration displacements can be considered as being sampled uniformly in the angular domain, which is referred to as synchronously sampled data. In other words, no matter what the RPM (Revolutions Per Minute) of the measured blade is, there will always be uniform I data samples per revolution. As we know, order analysis techniques can enable us to analyze vibration signals when the rotating speed changes over time, including basic order analysis [18] and order tracking analysis [25]. Therefore, order analysis techniques can provide a promising solution for BTT vibration monitoring under VRS. As mentioned before, a BTT system samples the data at constant angular increments and synchronous sampling is not needed, so existing computed order tracking methods are not considered in this paper. According to the order sampling theorem, the sampling number per revolution should be larger than twice the maximum EO of interest. That is to say, we need to mount at least 20 BTT sensors if we want to monitor the 10th EO. In practice, the number of BTT sensors is small, so the order sampling is also under-sampled in terms of high EOs of interest. In this case, we cannot directly carry out order analysis, and we also need to reconstruct high EOs of interest.

**Figure 2:**
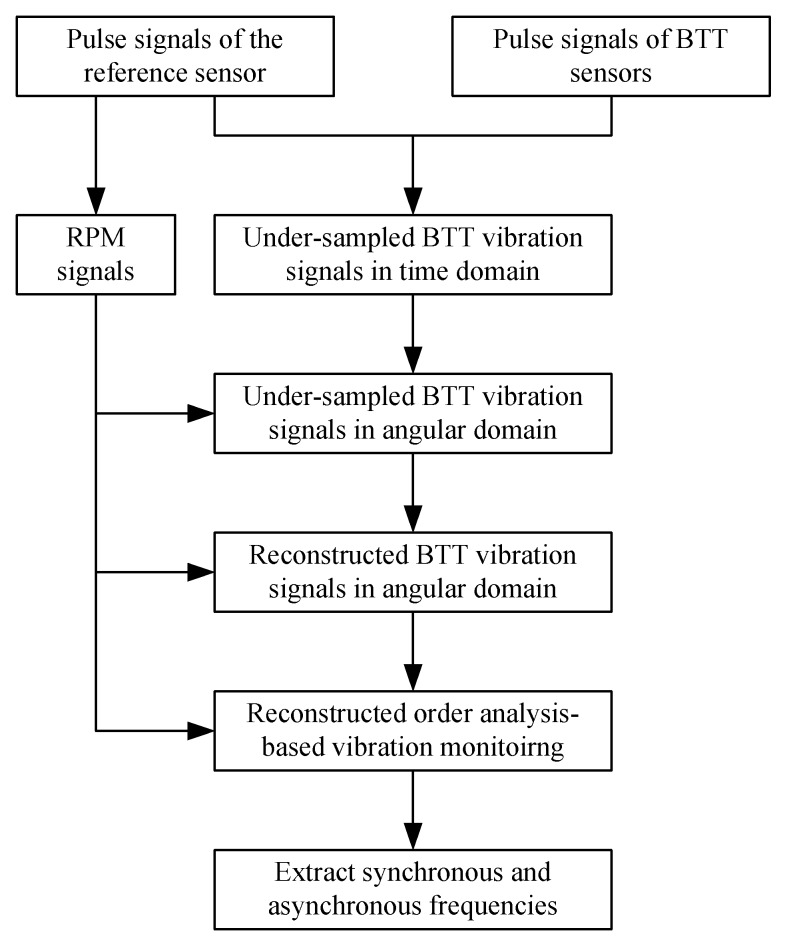
The structure of reconstructed order analysis-based BTT vibration monitoring.

In this section, we propose a basic structure of reconstructed order analysis-based BTT vibration monitoring, which is shown in Figure 2. There are three key steps. The first is to transform BTT vibration signals in the time domain into angularly sampled vibration signals. The second is to reconstruct under-sampled vibration signals in the angular domain. The third is to perform order analysis to extract characteristic blade vibration frequencies for vibration monitoring.

Furthermore, assuming that the rotating speed is a constant during each revolution, angularly sampled vibration displacements of the *k*th blade under VRS can be represented as:

(7) dk[l]=2πRT⌊lI⌋+1(t˜⌊lI⌋+1,k,mod(l,M)actual−t˜⌊lI⌋+1,k,mod(l,M)expected)=2πRT⌊lI⌋+1(t˜⌊lI⌋+1,k,mod(l,I)actual−αmod(l,I)−θk2π×T⌊lI⌋+1) 

where l denotes the *l*th sampled vibration displacement, ⌊x⌋ denotes the maximum integer less than *x*. mod(l,I) is the modulus of l divided by I and we define $\mathrm{mod}\left(qI,I\right)=q-1(q\in Z^{+})$ here.

## 3. Angular-Domain BTT Vibration Reconstruction Algorithms under VRS

### 3.1. Uniform BTT Sensor Configuration

To reconstruct any sampled signal in the analog domain, a sampling function can be used to perform weighted interpolation on discrete sampling points. For angularly sampled signals under uniform BTT sensor configuration, we can use this reconstruction process to recover continuous-angle signals. Similarly, the Shannon sampling theorem in the time domain can be extended as follows in angular domain.

Shannon sampling theorem in angular domain: The maximum order of sampled signal is denoted as ${\mathrm{EO}}_{\max}$. If the angular sampling frequency is larger than ${\mathrm{EO}}_{\max}/2$, then the continuous-angle signal can be reconstructed perfectly as Equation (8).

(8) $$\bar{y}\left(\theta \right)=\sum_{n=-\infty }^{\infty }y\left[k\right]\frac{\sin\pi \left({\mathrm{EO}}_{s}\times \theta -k\right)}{\pi \left({\mathrm{EO}}_{s}\times \theta -k\right)}\;$$

where y[k] is the angular sampling signal, ${\mathrm{EO}}_{s}$ is the angular sampling frequency and k is the number of angular samplings.

In practice, the number of BTT sensors is small, and EO of interest is always high, so that order aliasing will exist, and it is difficult to extract high EOs using Equation (8). However, for a high EO of interest, we can only focus on a band-pass signal z(θ) with center order ${\mathrm{EO}}_{0}$ and order bandwidth $E_{0}$, i.e., $\mathrm{EO}\in \left[{\mathrm{EO}}_{0}-E_{0}/2,{\mathrm{EO}}_{0}+E_{0}/2\right]$. Then an angular-domain BTT vibration reconstruction algorithm under uniform sensor configuration is proposed as follows based on the band-pass sampling theorem.

First, z(θ) is shifted by ${\mathrm{EO}}_{0}$ in the order domain and we will have

(9) $$s\left(\theta \right)=z\left(\theta \right)e^{-j2\pi {\mathrm{EO}}_{0}\theta }\;$$

So s(θ) becomes a low-pass signal with zero center order and bandwidth $E_{0}$. Let the angular sampling frequency in the BTT method be ${\mathrm{EO}}_{s}$. According to the above Shannon sampling theorem in angular domain, we can reconstruct s(θ) from the angular sampling signal s[k] as follows if only $E_{0}<{\mathrm{EO}}_{s}$.

(10) s^(θ)=Re[∑k=−∞∞s˜[k]sinc(EOs×θ−k)] 

where $\tilde{s}\left[k\right]$ is the analytic version of s[k].

It should be pointed out that here ${\mathrm{EO}}_{s}$ is just equal to the number of BTT sensors, thus, the criterion of choosing $E_{0}$ is to satisfy $E_{0}<I$. Furthermore, the band-pass signal z(θ) can be reconstructed as:

(11) z^(θ)=Re[∑n=−∞∞s˜[k]sinc(EOs×θ−k)exp(j2πEO0EOs(EOs×θ−k))] 

It can be seen from Equation (10) that the band-pass signal z(θ) can be reconstructed without error from the sub-sampled signal s[k]. That is to say, it does not need to satisfy ${\mathrm{EO}}_{s}>2{\mathrm{EO}}_{0}+E_{0}$, so that we can use few BTT sensors to extract high EO in blade vibrations. Here, how to choose $E_{0}$ depends on the practical requirement. Assuming that the frequency band of interest is $\left[f_{\min},f_{\max}\right]$, the corresponding order band can be chosen as $\left[{\mathrm{EO}}_{\min},{\mathrm{EO}}_{\max}\right]$,

(12) $${\mathrm{EO}}_{\min}=\left[f_{\min}/f_{\max}^{r}\right],\;{\mathrm{EO}}_{\max}=\left[f_{\max}/f_{\min}^{r}\right]\;$$

where ‘[ ]’ is the integer operator and $f_{\min}^{r},f_{\max}^{r}$ are the minimum and maximum blade rotating frequencies, respectively. In this case, $E_{0}$ can be chosen as:

(13) $$E_{0}=\frac{{\mathrm{EO}}_{\max}+{\mathrm{EO}}_{\min}}{2}\;$$

The order width $B_{\mathrm{EO}}$ can be calculates as:

(14) $$B_{EO}={\mathrm{EO}}_{\max}-{\mathrm{EO}}_{\min}\;$$

It is obvious that the reconstruction performance depends crucially on the choice of the interpolation function. The sinc function in Equation (11) decays very slowly, so its calculation often consumes significant time. Many other functions have been studied thoroughly in sampling. In particular, there are several advantages to using splines for reconstruction. First, the best kernels that are able to achieve minimum approximation error can be expressed using derivatives of B-splines of approximation order. Second, spline functions have the minimum support among all the functions with the same order of approximation. Third, B-splines are easily and straightforwardly used in the discrete domain. Therefore, an optimal reconstruction kernel function $K_{B}\left(\theta \right)$ based on six-order B-Splines is applied here to replace the sinc function. Then Equation (11) can be rewritten as:

(15) z^(θ)=Re[∑n=−∞∞s˜[k]KB(EOs×θ−k)exp(j2πEO0EOs(EOs×θ−k))] 

In the end, it should be emphasized that the center order ${\mathrm{EO}}_{0}$ and order bandwidth $E_{0}$ are not arbitrarily selected. The aim of the proposed method is to reconstruct orders among $\left[{\mathrm{EO}}_{0}-E_{0}/2,{\mathrm{EO}}_{0}+E_{0}/2\right]$, so ${\mathrm{EO}}_{0}$ and $E_{0}$ should be selected to make each EO of interest satisfy $\mathrm{EO}\in \left[{\mathrm{EO}}_{0}-E_{0}/2,{\mathrm{EO}}_{0}+E_{0}/2\right]$. Furthermore, in practice, we need to estimate EOs of interest related to damages in advance by using finite element analysis or experimental testing. In this case, any order among $\left[{\mathrm{EO}}_{0}-E_{0}/2,{\mathrm{EO}}_{0}+E_{0}/2\right]$ can be reconstructed for blade vibration monitoring, including synchronous and asynchronous vibrations. Otherwise, they cannot be detected.

### 3.2. Non-Uniform BTT Sensor Configuration

In some cases, ideal uniform BTT sensor configuration may not be carried out due to space limitation or/and installation errors, which will bring reconstruction errors into Equation (11). Fortunately, even for I non-uniform BTT sensors, it can still be considered as a uniform configuration for each BTT sensor. That is to say, the angular sampling interval of each sensor is equal to 2π. Based on that, a new angular-domain reconstruction algorithm for non-uniform BTT sensor configuration is proposed as follows:

Let us assume that I BTT sensors are mounted non-uniformly. In this case, if the rotation speed is variable, the non-uniform BTT sampling function of blade vibrations in the angular domain can be formulated as follows:

(16) $$\bar{d}(\theta )=\sum_{i=0}^{I-1}\sum_{n=-\infty }^{\infty }d(\theta +\alpha_{i})\delta (\theta -2\pi n)\;$$

where d(θ) is the true vibration signal in the angular domain and δ(θ) is the Dirac delta function.

Furthermore, it can be seen from Equation (16) that non-uniformly angular sampling signal is equivalent to the sum of I uniform sample streams. $\alpha_{i}$ can be looked at as the angle-offset of the *i*th sample stream. The order spectrum of $\bar{d}\left(\theta \right)$ is

(17) $$\bar{D}\left(EO\right)=\frac{1}{2\pi }\sum_{i=0}^{I-1}D\left(\mathrm{EO}\right)e^{j2\pi \alpha_{i}\mathrm{EO}}⊗\sum_{n=-\infty }^{\infty }\delta \left(\mathrm{EO}-n\right)=\frac{1}{2\pi }\sum_{i=0}^{I-1}\left[\sum_{n=-\infty }^{\infty }D\left(\mathrm{EO}-n\right)e^{-j2\pi \alpha_{i}n}\right]e^{j2\pi \alpha_{i}\mathrm{EO}}\;$$

where ⊗ is the convolution operator.

Based on Equation (17), we can see that a finite number of replicas intersect with the region $\left[{\mathrm{EO}}_{\min},{\mathrm{EO}}_{\max}\right]$ in the order spectrum of each sample stream. To interpolate D(EO), it is necessary to prevent aliasing in the reconstructed signal. That is to say, all unwanted replicas of D(EO) in the region $\left[{\mathrm{EO}}_{\min},{\mathrm{EO}}_{\max}\right]$ must be removed. This can be achieved by using interpolation function for each sample stream such that all unwanted replicas sum to zero. Based on the signal reconstruction theory, the reconstruction of d(θ) can be formulated as:

(18) $$\hat{d}(\theta )=\sum_{i=0}^{I-1}\sum_{n=-\infty }^{\infty }d\left(2\pi n+\alpha_{i}\right)h_{i}\left(\theta -2\pi n\right)\;$$

where $h_{i}(\theta )$ is the *i*th interpolating function for the *i*th sample stream (i.e., the *i*th BTT sensor).

On the other hand, these unwanted replicas can be selected by truncating the sum of Equation (17) such that

(19) $${\bar{D}}_{T}\left(\mathrm{EO}\right)=\frac{1}{2\pi }\sum_{i=0}^{I-1}\left[\sum_{n=n_{\min}}^{n_{\max}}D\left(\mathrm{EO}-n\right)e^{-j2\pi \alpha_{i}n}\right]e^{j2\pi \alpha_{i}\mathrm{EO}}\;$$

where $n_{\min}$, $n_{\max}$ are integers which are defined as follows:

$$n_{\min}=\min\left(n\right)\;\mathrm{such}\;\mathrm{that}\;{\mathrm{EO}}_{\max}+n>{\mathrm{EO}}_{\min},\;i.e.,\;n_{\min}=\left\lceil {\mathrm{EO}}_{\min}-{\mathrm{EO}}_{\max}\right\rceil$$

$$n_{\max}=\max\left(n\right)\;\mathrm{such}\;\mathrm{that}\;{\mathrm{EO}}_{\min}+n<{\mathrm{EO}}_{\max},\;i.e.,\;n_{\max}=\left\lfloor {\mathrm{EO}}_{\max}-{\mathrm{EO}}_{\min}\right\rfloor \;$$

where ⌊x⌋ denotes the most integer no more than *x* and ⌈x⌉ denotes the smallest integer no less than *x*.

Next, the key problem is how to calculate the interpolating function $h_{i}\left(\theta \right)$. But it is difficult to directly derive analytical solutions of interpolating functions in the angular domain. In nature, the target is to use interpolating functions to remove all unwanted replicas in the region $\left[{\mathrm{EO}}_{\min},{\mathrm{EO}}_{\max}\right]$, such that $\bar{D}\left(\mathrm{EO}\right)=\hat{D}\left(\mathrm{EO}\right)$. Then one can solve the above problem in the order domain. To achieve it, the ranges of $\left[{\mathrm{EO}}_{\min},{\mathrm{EO}}_{\max}\right]$ are separated into a number of sub-bands and each sub-band has different intersected parts of replicas [32]. In each sub-band, simultaneous equations of the following form are built to ensure that all unwanted replicas sum to zero by combining Equation (18) and Equation (19).

(20) $$\frac{1}{2\pi }\sum_{i=0}^{I-1}e^{-j2\pi \alpha_{i}n}e^{j2\pi \alpha_{i}\mathrm{EO}}H_{i}(\mathrm{EO})e^{-jk\alpha_{i}}=\delta \left(n\right),n\in \left[n_{\min},n_{\max}\right]\;$$

where $H_{i}\left(\mathrm{EO}\right)$ is the order spectrum of $h_{i}(\theta )$.

Finally, $H_{i}\left(\mathrm{EO}\right)$ can be solved based on Equation (20), leading to the solution of $h_{i}\left(\theta \right)$. It should be noted that the number of sub-bands increases with I and $B_{\mathrm{EO}}$. In this case, large I or/and $B_{\mathrm{EO}}$ will make it difficult to solve Equation (20). On the other hand, fewer BTT sensors are expected in practice due to some limitations. Thus, here two BTT sensors are considered, i.e., I=2. Without loss of generality, $\alpha_{0}$ is assumed to be zero. Then the corresponding second-order reconstruction formula can be solved as follows by referring to the idea in Reference [33].

(21) $$\hat{d}(\theta )=\sum_{n=-\infty }^{\infty }\left\{d\left(\frac{n}{E_{0}}\right)h\left(\theta -\frac{n}{E_{0}}\right)+d\left(\frac{n}{E_{0}}+\frac{\alpha_{1}}{2\pi E_{0}}\right)h\left(-\theta +\frac{n}{E_{0}}+\frac{\alpha_{1}}{2\pi E_{0}}\right)\right\}\;$$

where

(22) $$\begin{array}{l} h\left(\theta \right)=\frac{\cos\left[2\pi \left(mE_{0}-{\mathrm{EO}}_{\min}\right)\theta -m\alpha_{1}/2\right]-\cos\left(2\pi {\mathrm{EO}}_{\min}\theta -m\alpha_{1}/2\right)}{2\pi E_{0}\theta \sin\left(m\alpha_{1}/2\right)}\\ +\frac{\cos\left[2\pi \left(E_{0}+{\mathrm{EO}}_{\min}\right)\theta -\left(m+1\right)\alpha_{1}/2\right]-\cos\left[2\pi \left(mE_{0}-{\mathrm{EO}}_{\min}\right)\theta -\left(m+1\right)m\alpha_{1}/2\right]}{2\pi E_{0}\theta \sin\left[\left(m+1\right)\alpha_{1}/2\right]} \end{array}$$

where $m=\left\lceil 2{\mathrm{EO}}_{\min}/E_{0}\right\rceil$. At the same time, it must be emphasized that there are two important costraints in Equation (22), i.e., $\sin(m\alpha_{1}/2)\neq 0$ and $\sin((m+1)\alpha_{1}/2)\neq 0$. Once these constraints cannot be satisfied, orders of interest will not be achieved. Therefore, the angular positions of two BTT sensors should be selected carefully in engineering applications.

## 4. Numerical Simulations and Validations

### 4.1. Simulated Vibration Signals with Variable Frequencies

It is assumed that the rotating speed increases from $r_{L}$ to $r_{H}$ during *N* revolutions and two synchronous blade vibrations are generated. The corresponding engine orders are denoted as ${\mathrm{EO}}_{1}$ and ${\mathrm{EO}}_{2}$, respectively. Then each resonant frequency is calculated as follows:

(23) $$f_{i}={\mathrm{EO}}_{i}\times \left(r_{L}+\frac{\left(n-1\right)K}{2}\right)/60\;$$

where $K=\left(r_{H}-r_{L}\right)/N$, i=1,2, n=1,…,N.

Then a multi-frequency signal is simulated as follows:

(24) $$x\left(t\right)=A_{1}\sin\left(2\pi f_{1}t+\varphi_{1}\right)+A_{2}\sin\left(2\pi f_{2}t+\varphi_{2}\right)+A_{3}\sin\left(2\pi f_{3}t+\varphi_{3}\right)\;$$

where $f_{1}$ and $f_{2}$ are the two resonant vibration frequencies and $f_{3}$ is an asynchronous vibration frequency. Here, the simulation parameters are chosen as: *N* = 1000, $r_{L}=5000$ RPM, $r_{H}=5625$ RPM, ${\mathrm{EO}}_{1}=8$, ${\mathrm{EO}}_{2}=9$, $f_{3}=750$ Hz, $A_{1}=1$ mm, $A_{2}=0.5$ mm, $A_{3}=1$ mm, and $\varphi_{1}=\varphi_{2}=\varphi_{3}=0$.

**Figure 3:**
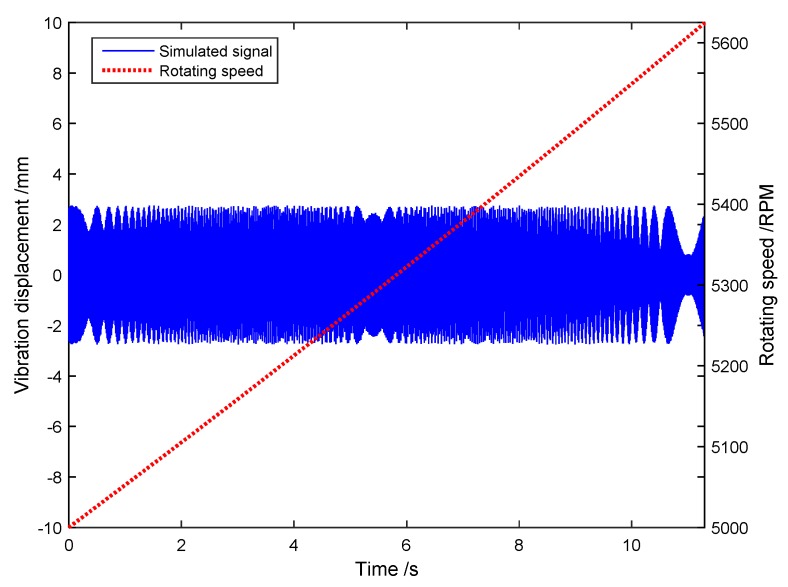
The waveforms of both the original signal and the rotating speed.

**Figure 4:**
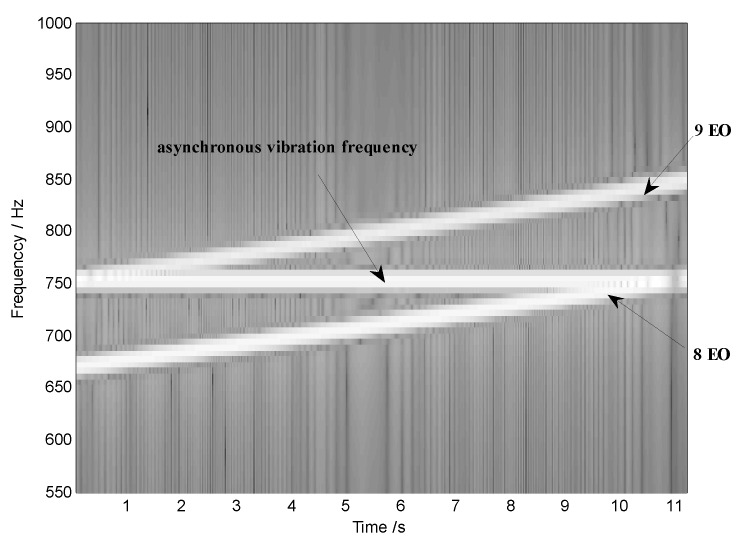
Short-time Fourier transform (STFT) of the original signal.

Then the waveforms of both x(t) and the rotating speed are shown in Figure 3. Furthermore, the short-time Fourier transform (STFT) of the original signal is plotted as Figure 4, where we can see two time-variable frequency components and a constant frequency one.

### 4.2. Reconstruction Results under Uniform BTT Sensor Configuration

**Figure 5:**
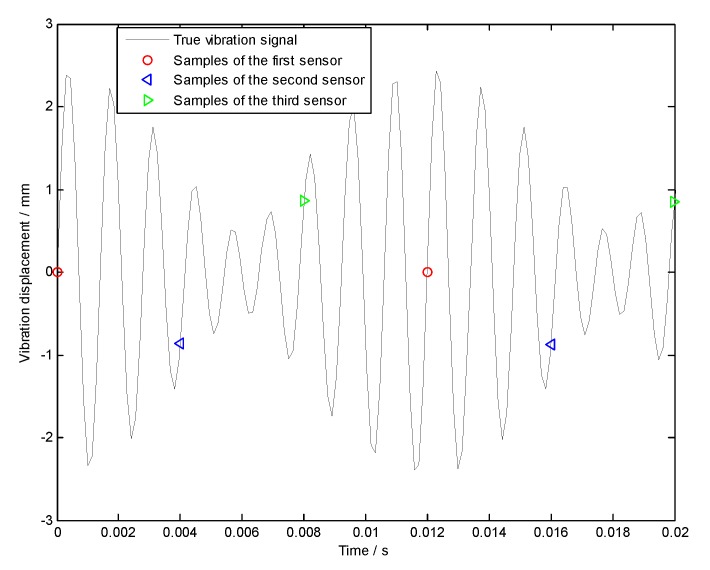
Uniformly sampled signals of three BTT sensors.

For uniform BTT sensor configuration, three BTT sensors are mounted uniformly around the circumference. That is to say, the angular interval of two adjacent blades is equal to 120 degree. Under variable rotation speeds, the period of each revolution can be calculated by using measured instantaneous rotating speeds. Based on it, the sampling times of each BTT sensor can be estimated. Then we can get the sampled vibration signals of three BTT sensors by substituting the sampling times into Equation (24) and the corresponding BTT sampling signals are shown in Figure 5.

**Figure 6:**
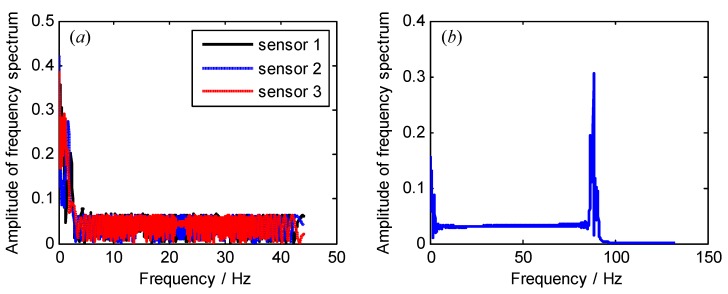
Frequency spectrums of sampled signals from both (**a**) each BTT sensor and (**b**) three BTT sensors.

Frequency spectrums of sampled signals from both each BTT sensor and three BTT sensors are shown in Figure 6. Obviously, we cannot see the resonant and asynchronous frequencies. The reasons may include: (i) The maximum sampling frequency is 281.25 Hz, so the sampled signals are under-sampled, and the corresponding power spectrums are not true; and (ii) the rotating speed is variable, so the sampled signals tend to be non-stationary. In this case, their frequency spectrums are also not accurate. Next, the angular-domain BTT vibration reconstruction algorithm in Section 3.1 is used to extract the true vibration frequencies.

**Figure 7:**
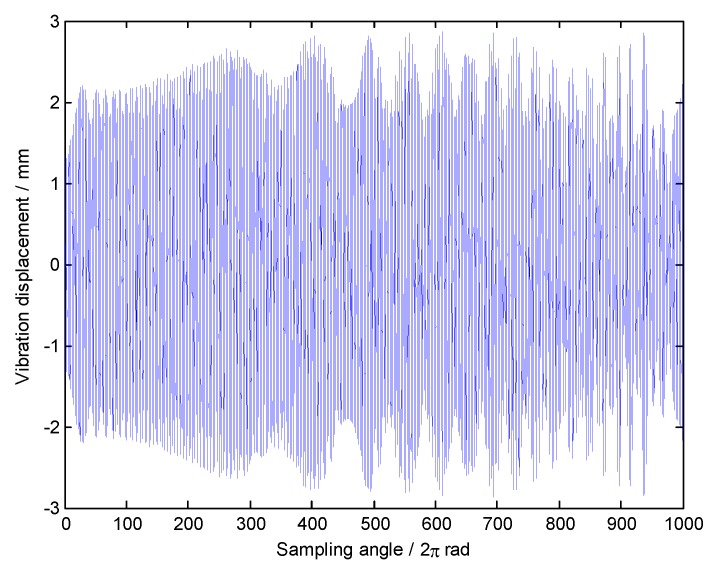
The angular-domain reconstruction signal under variable rotation speed (VRS).

**Figure 8:**
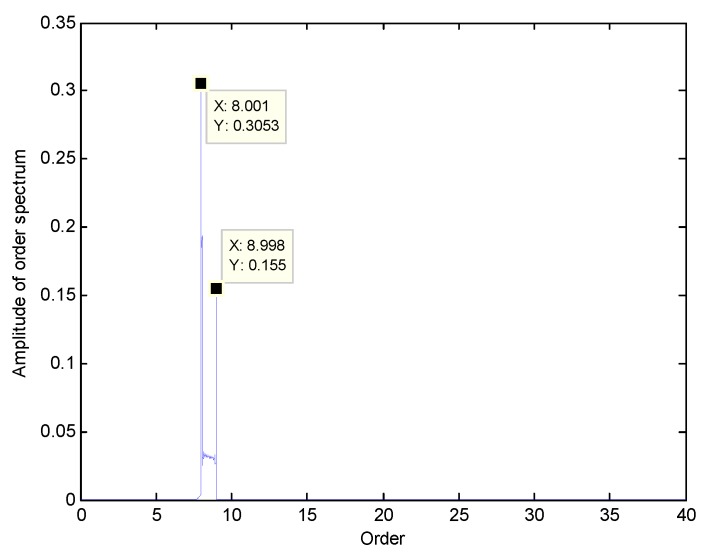
Order spectrum of the angular-domain reconstruction signal.

To apply Equation (11), the center order ${\mathrm{EO}}_{0}$ and order bandwidth $E_{0}$ should be selected in advance. Here, ${\mathrm{EO}}_{s}=3$ and the orders of interest are ${\mathrm{EO}}_{1}=8$, ${\mathrm{EO}}_{2}=9$. In this case, we choose $E_{0}=2$, so that ${\mathrm{EO}}_{s}>E_{0}$. As mentioned before, the selection criterion of ${\mathrm{EO}}_{0}$ is to satisfy ${\mathrm{EO}}_{1},{\mathrm{EO}}_{2}\in \left[{\mathrm{EO}}_{0}-E_{0}/2,{\mathrm{EO}}_{0}+E_{0}/2\right]$. Here we choose ${\mathrm{EO}}_{0}=8.5$ as an example. In addition, θ in Equation (11) is a continuous-time variable, so it needs to be discretized for engineering applications. Therefore, a re-sampling frequency ${\mathrm{EO}}_{res}$ in angular domain is used so that the Shannon sampling theorem is satisfied, namely ${\mathrm{EO}}_{res}>2{\mathrm{EO}}_{0}+E_{0}$. Here we choose ${\mathrm{EO}}_{res}=80$. The angular-domain reconstruction signal of the under-sampled BTT signal under VRS is shown in Figure 7. Furthermore, its Fourier transform was calculated, and the order spectrum is shown in Figure 8. Obviously, we can see the 8th EO and the 9th EO, so two synchronous vibration frequencies are accurately reconstructed.

**Figure 9:**
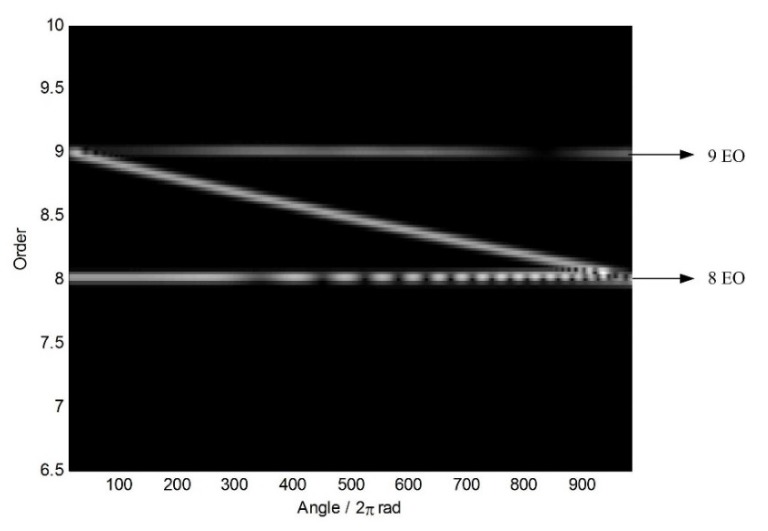
Time-varying order spectrum of the angular-domain reconstruction signal.

**Figure 10:**
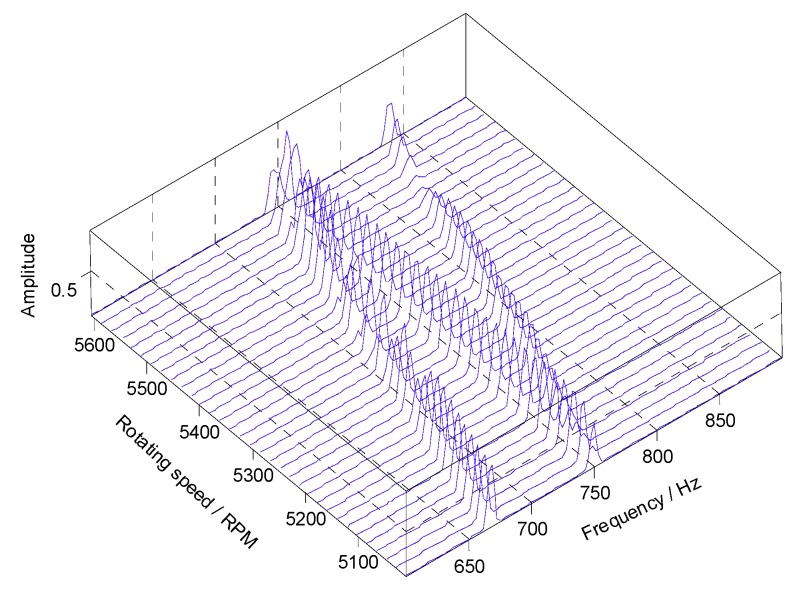
Waterfall of the angular-domain reconstruction signal based on STFT.

At the same time, however, there are some vague orders between the 8th EO and the 9th EO in Figure 8. To explore these non-stationary components, STFT of the angular-domain reconstruction signal is calculated, and the time-varying order spectrum is shown in Figure 9. Interestingly, there is an order line which means that the order changes linearly with the angle, besides of the 8th EO and the 9th EO. To analyze this line, we can calculate the rotating frequency at each angular sampling point by using the curve of rotating speed shown in Figure 3. Then, the vibration frequency of each sampling point can be obtained by multiplying its rotating frequency and the corresponding order shown in Figure 9. Based on it, the waterfall of the angular-domain reconstruction signal is calculated as Figure 10 based on STFT. We can see that: (i) There are two frequency components which change linearly with the rotating speed, which are just the two EOs; and (ii) there is a constant frequency component at about 750 Hz, which is just equal to the asynchronous vibration frequency $f_{3}$. The above results testify that the proposed algorithm can recover characteristic vibration frequencies from under-sampled BTT vibration signals under uniform sensor configuration and VRS.

### 4.3. Reconstruction Results under Non-Uniform BTT Sensor Configuration

**Figure 11:**
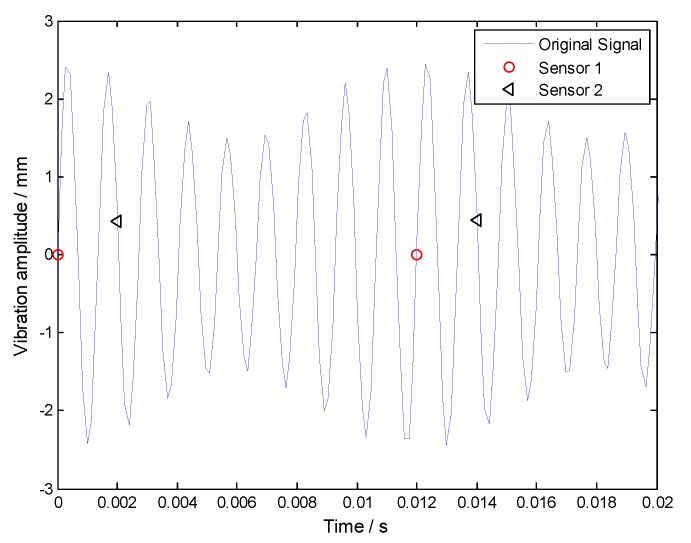
Non-uniformly sampled signals of two BTT sensors in the time domain.

To validate the proposed reconstruction algorithm in Section 3.2, two BTT sensors are assumed to be used, and their angular interval is equal to 60 degrees. Similarly, we can calculate the sampling times, which are substituted into Equation (24) to get the sampled vibration signals of two BTT sensors. Finally, the simulated BTT vibration signals are shown in Figure 11.

**Figure 12:**
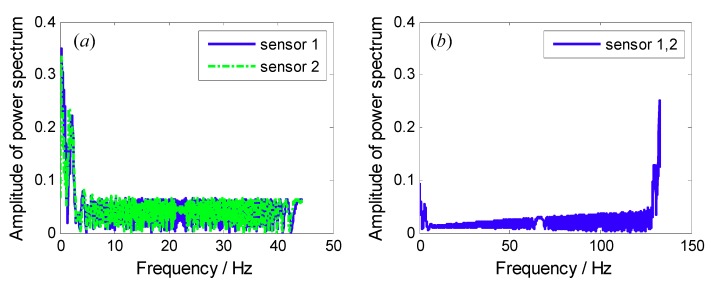
Frequency spectrums of sampled signals from both (**a**) each BTT sensor and (**b**) two BTT sensors.

Frequency spectrums of sampled signals from both each BTT sensor and two BTT sensors are shown in Figure 12. Similarly, we cannot see the synchronous and asynchronous frequencies. To overcome this problem, the proposed algorithm in Section 3.2 will be used to reconstruct the vibration signal in the angular domain.

**Figure 13:**
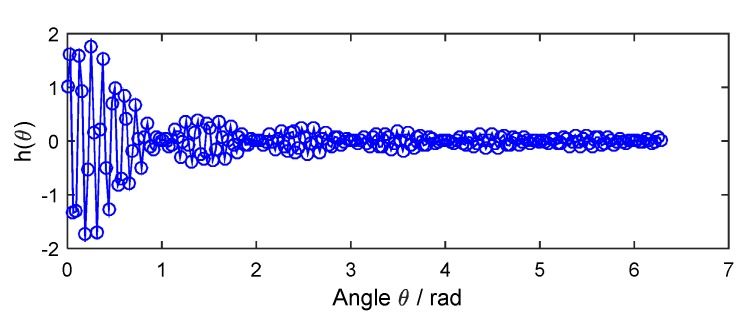
The interpolation function in Equation (22).

**Figure 14:**
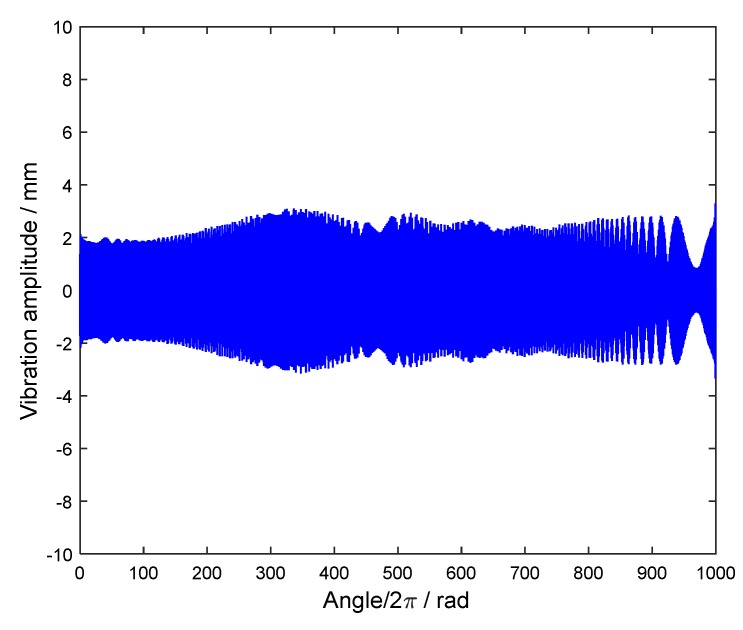
The angular-domain reconstruction signal of the under-sampled BTT signal using two BTT sensors under VRS.

**Figure 15:**
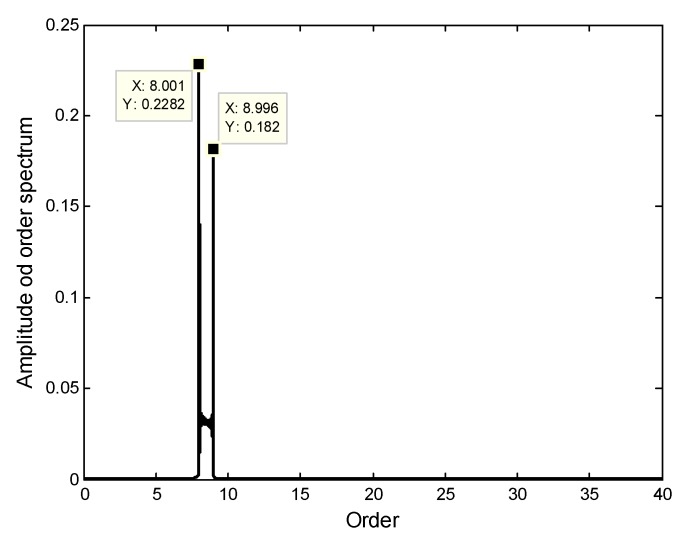
Order spectrum of the angular-domain reconstruction signal using two non-uniform BTT sensors under VRS.

Here $E_{0}$ and $\alpha_{1}$ in Equation (21) are chosen as $E_{0}=2$ and $\alpha_{1}=\pi /3$, respectively. The interpolation function h(θ) in Equation (22) is estimated and shown in Figure 13. Based on it, the angular-domain reconstruction signal of the under-sampled BTT signal under VRS is shown in Figure 14. Furthermore, its Fourier transform is calculated, and the order spectrum is shown in Figure 15. We can clearly see that Figure 15 is almost the same as Figure 8 and two synchronous vibration frequencies are also accurately reconstructed.

**Figure 16:**
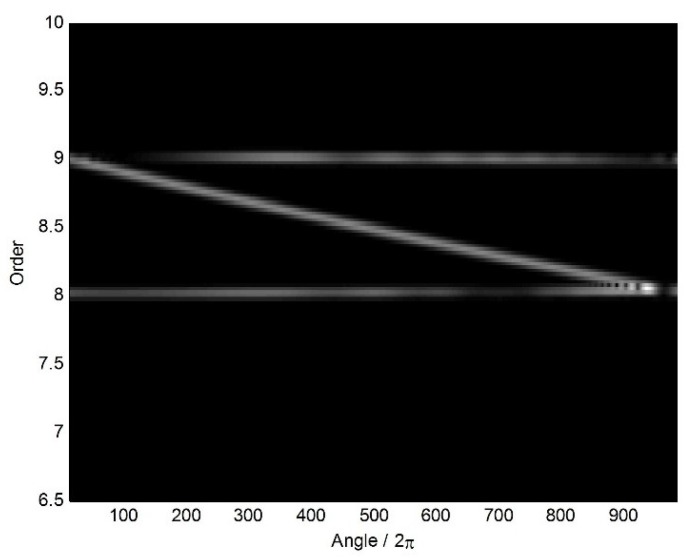
Time-varying order spectrum of the angular-domain reconstruction signal using two non-uniform BTT sensors under VRS.

**Figure 17:**
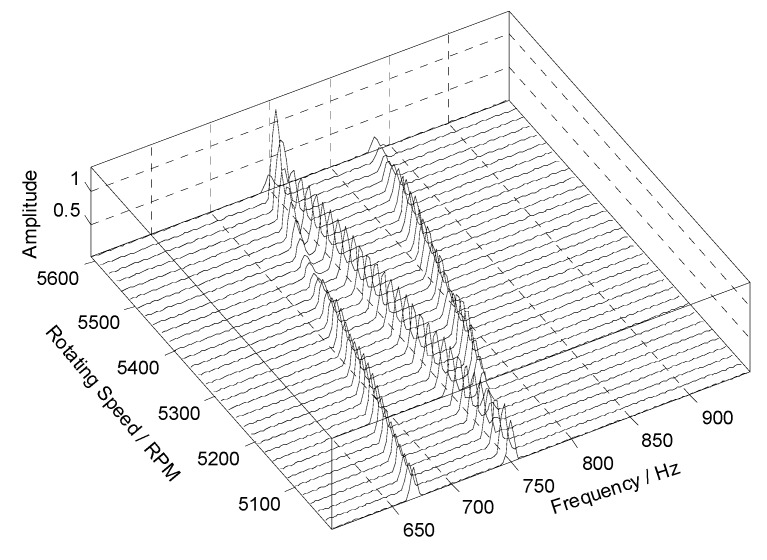
Waterfall of the angular-domain reconstruction signal using two non-uniform BTT sensors under VRS.

Furthermore, the time-varying order spectrum and waterfall of the angular-domain reconstruction signal from two non-uniform BTT sensors are shown in Figure 16 and Figure 17, respectively. Compared with Figure 9 and Figure 10, we make a similar conclusion that characteristic vibration frequencies can be reconstructed under non-uniform sensor configuration and VRS. Therefore, the proposed algorithm is validated.

Here, it should be emphasized that the simulated BTT signals are well under-sampled, so it is impossible to accurately detect the vibration amplitude associated with each order. That is to say, only the order values are reliable. However, it is enough to perform on-line blade vibration monitoring.

## 5. Experimental Validations

### 5.1. Experimental Set-Up

**Figure 18:**
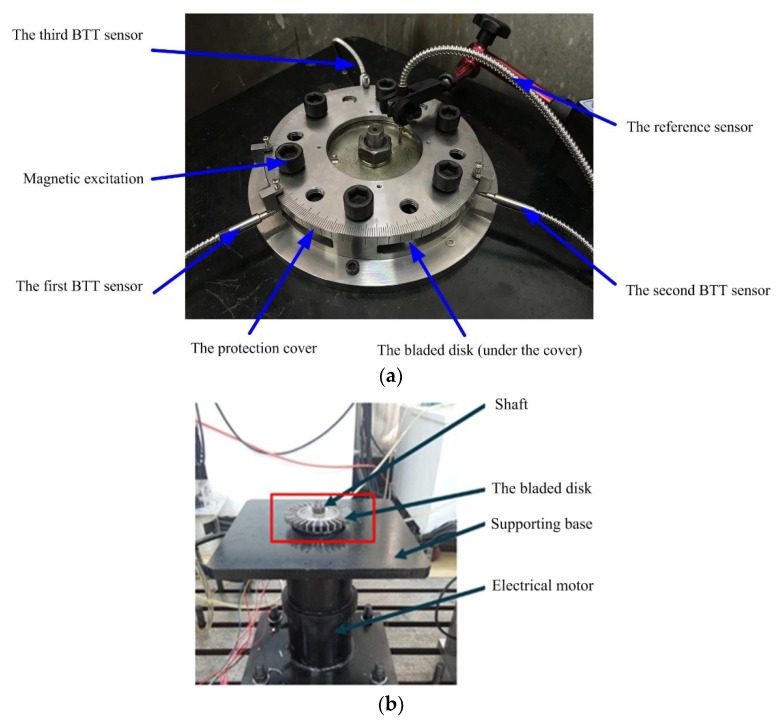
Experimental rig of high-speed rotating blades. (**a**) The whole view; (**b**) Uncovered view.

To further validate the proposed BTT vibration reconstruction algorithms, an experimental rig of BTT-based vibration monitoring of high-speed rotating blades was built as Figure 18. The whole experimental system was composed of a supporting base, an electrical motor, a testing bladed disk, a magnetic exciter, three optical fiber BTT sensors, a once-per-revolution reference sensor, and a protection cover. The electrical motor was mounted under the supporting base. BTT sensors were fixed around the bladed disk through the holes in the protection cover. An angular scale was presented on the surface of the protection cover, so that the angle between two BTT sensors could be easily and accurately adjusted. The reference sensor was mounted near the center of the bladed disk. There were 32 blades on the bladed disk, which were labeled from 1 to 32. Mechanical and geometrical features of blades are presented in Table 1.

Vibration excitations were generated by six permanent magnets mounted near the blades, and different configurations of permanent magnets can result in different excitations. A BTT vibration measurement system was designed to collect BTT signals for analysis and validation.

To evaluate experimental results, it needs to identify a blade’s synchronous vibration parameters in advance. First, natural frequency of a blade was estimated as 2140.3 Hz by finite element analysis (ANSYS software). Second, the sweep-frequency fitting method in Reference [26] was used to monitor the variations of vibration displacements with the rotating speed, and then synchronous resonance regions of the blade was obtained as 61–65 Hz, 73–78 Hz, 92–97 Hz, and 124–129 Hz, respectively. Finally, based on the central frequencies of resonant regions, the global autoregressive with instrumental variables (GARIV) method in Reference [27] was used to identify the corresponding EOs as 36, 30, 24, and 18. In the following experiments, two different configurations of permanent magnets were used to excite different resonant blade vibrations. Then BTT signals were collected to calculate under-sampled blade vibrations and the proposed algorithms were used to reconstruct the expected EOs.

### 5.2. Experimental Results under Uniform Sensor Configuration

**Figure 19:**
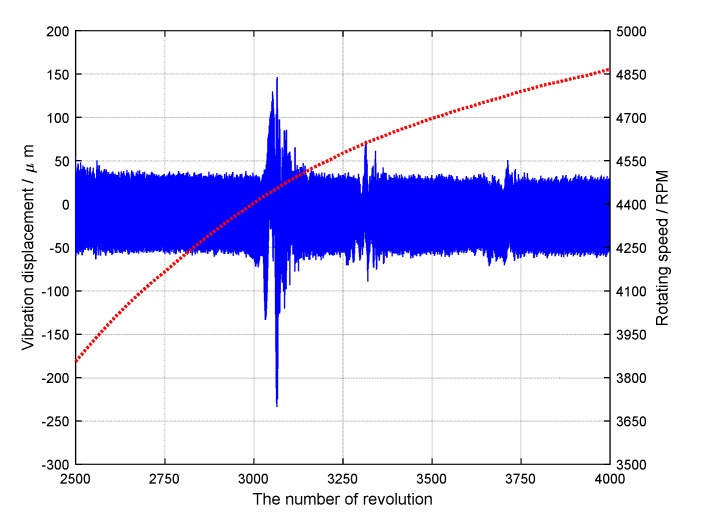
The angularly sampled vibration displacement and the rotating speed of the 24th blade.

In the first experiment, three BTT sensors were mounted uniformly around the bladed disk. Experimental data during 1000 revolutions were sampled, including BTT signals of 32 blades and the rotating speed. The reference sensor was a once-per-revolution sensor, so it was difficult to obtain the instantaneous rotating speed of each blade at each revolution. To solve this problem, one-dimensional interpolation was used to estimate the instantaneous rotating speed of each blade at each revolution based on the average rotating speed of each revolution. Next blade vibrations in the angular domain could be calculated based on instantaneous rotating speeds and BTT signals. Here the 24th blade was taken as an example. BTT data set between the 2500th and 4000th revolution were used for vibration analysis. In the experiment, the angularly sampled vibration displacement and the rotating speed of the 24th blade are shown in Figure 19. Obviously, the rotating speed was variable. It can be seen that vibration displacement changes greatly between the 3000th and 3200th revolution. The reason may be that high-order synchronous vibrations happen due to the magnetic excitations. Furthermore, the corresponding range of rotating frequency was 73.4 Hz–75.8 Hz. According to prior knowledge, we can infer that the EO of this synchronous vibration may be equal to 30. To validate it, the proposed EO reconstruction algorithm in Section 3.1 will be used to analyze the sampled BTT signals.

**Figure 20:**
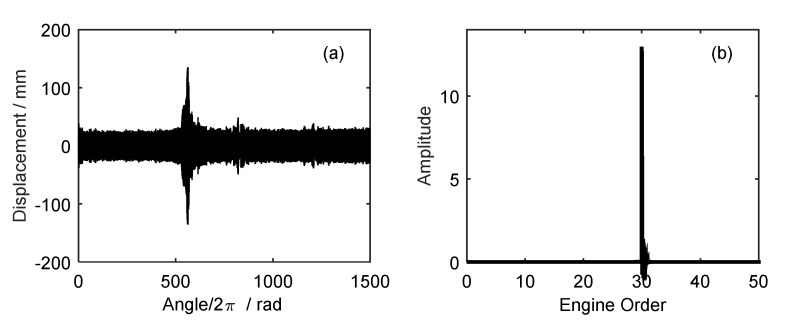
(**a**) The angular-domain reconstruction signal and (**b**) its order spectrum of the 24th blade.

Here three uniform BTT sensors were mounted, so the sampling order ${\mathrm{EO}}_{s}$ was also equal to three. As the same selection criterion mentioned before, here we chose $E_{0}=2$ and ${\mathrm{EO}}_{0}=30$. Then the sampled BTT vibration signals were processed by the Hilbert transform to obtain the analytic signals. Based on it, vibration signals of the 24th blade were angularly reconstructed by Equation (11) and then the order spectrum was calculated, both of which are shown in Figure 20. We can clearly see the 30th EO in the order spectrum as expected.

**Figure 21:**
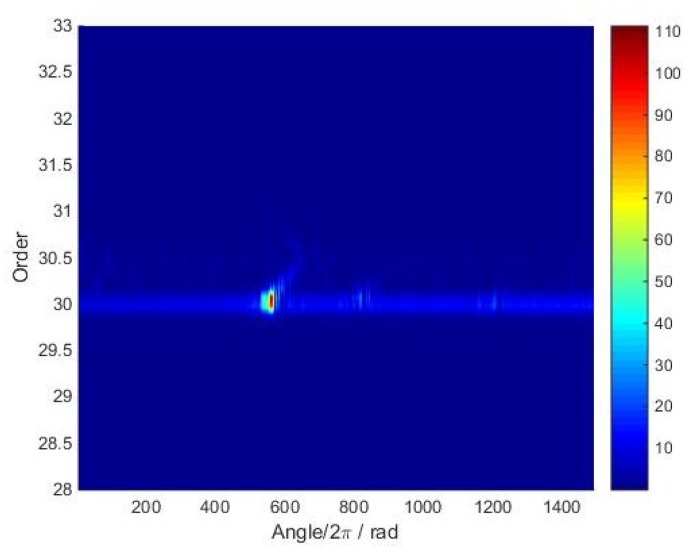
Time-varying order spectrum of the angular-domain reconstruction signal of the 24th blade.

**Figure 22:**
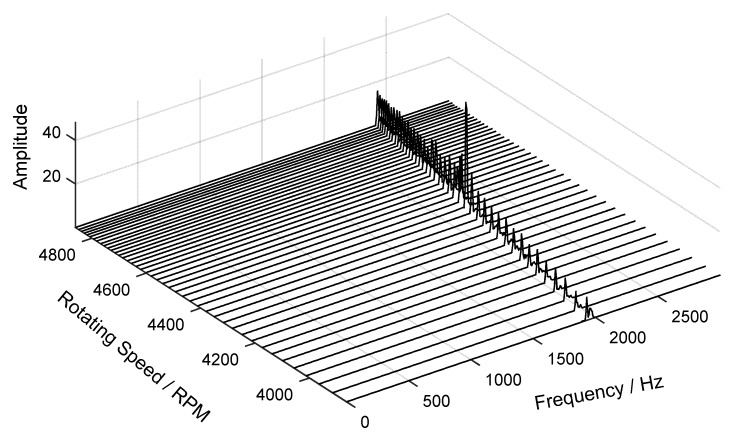
Waterfall of the angular-domain reconstruction signal of the 24th blade.

Next, the time-varying order spectrum of the angular-domain reconstruction signal was calculated by using the STFT method and is shown in Figure 21. It can be seen that the maximum vibration amplitude occurs at about the 560th revolution. Moreover, the waterfall of the angular-domain reconstruction signal is shown in Figure 22. The results show that vibration displacement of the 24th blade reaches the maximum value when the rotating speed is equal to 4440 RPM.

**Figure 23:**
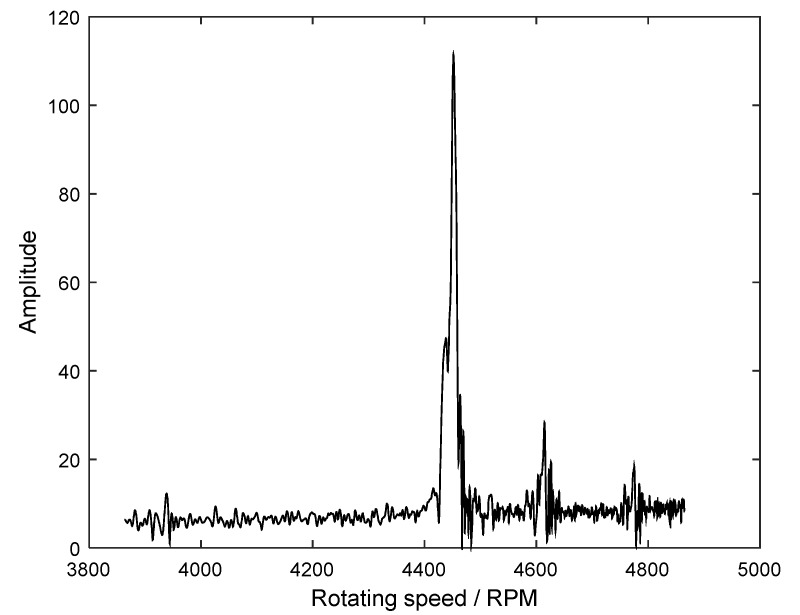
Order tracking spectrum of the 30th EO.

The exact resonant frequency could not be extracted in the waterfall, so the order tracking spectrum of the 30th EO was calculated and shown in Figure 23. From it, we can see that the 30th EO resonant vibration happens at the rotating speed of 4452 RPM. In this case, the corresponding resonant frequency was calculated as 2226 Hz. At the same time, the resonant frequency identified by the GARIV method was equal to 2270 Hz. Thus, the estimation error is only 1.9%.

### 5.3. Experimental Results under Non-Uniform Sensor Configuration

**Figure 24:**
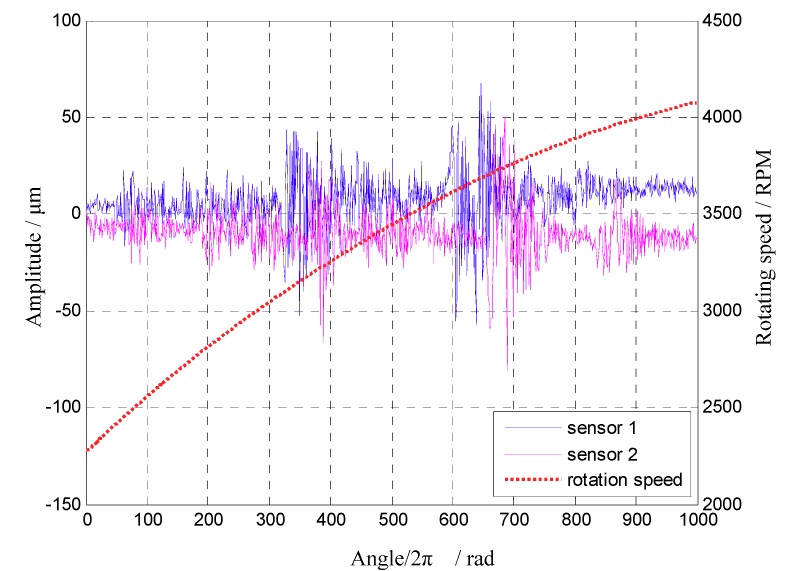
The angularly sampled vibration displacements and the rotating speed of the 11th blade.

In the second experiment, two BTT sensors were mounted, and the angular interval was equal to 77.4 degree so they are non-uniform. At the same time, a once-per-revolution reference sensor was used to sample the rotating speed. Here the 11th blade was taken as an example. BTT data sets of 1000 revolutions were used for vibration analysis. In the experiment, the angularly sampled vibration displacement and the rotating speed of the 11th blade are shown in Figure 24. Similarly, the rotating speed was variable.

**Figure 25:**
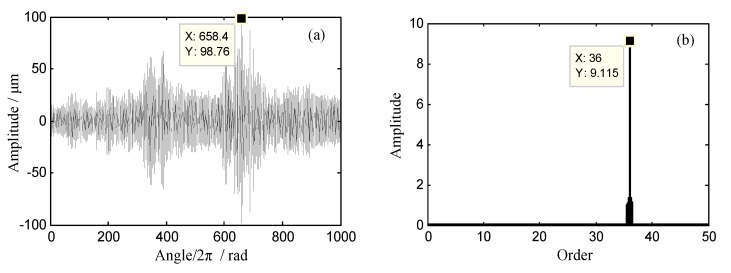
(**a**)The angular-domain reconstruction signal and (**b**) its order spectrum of the 11th blade.

It can be seen in Figure 24 that the vibration displacement changes greatly between the 600th and 700th revolution. Furthermore, the corresponding range of rotating frequency was 60 Hz–64 Hz. According to prior knowledge, we can infer that the EO of this synchronous vibration may be equal to 36. To validate it, the proposed EO reconstruction algorithm in Section 3.2 will be used to analyze the sampled BTT signals. The angular-domain reconstruction signal and its order spectrum of the 11th blade are shown in Figure 25. It can be seen that the 36th EO appears in the order spectrum and the maximum vibration amplitude occurs at about the 658th revolution.

**Figure 26:**
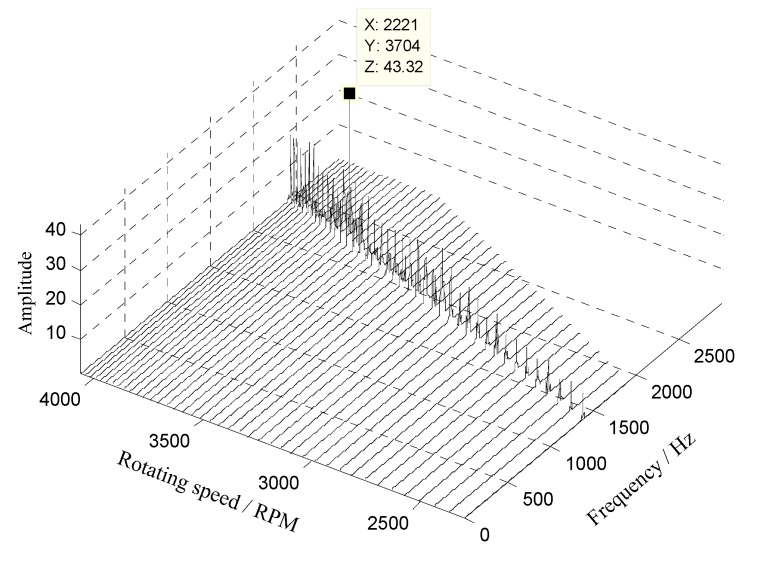
Waterfall of the angular-domain reconstruction signal of the 11th blade.

Next, the waterfall of the angular-domain reconstruction signal is shown in Figure 26. The results show that vibration displacement of the 11th blade reaches the maximum value when the rotating speed is equal to 3700 RPM.

**Figure 27:**
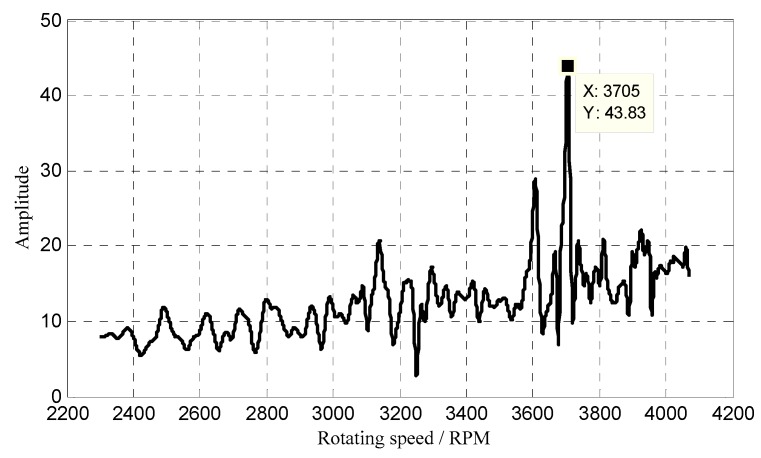
Order tracking spectrum of the 36th EO.

Furthermore, to seek the exact resonant frequency, the order tracking spectrum of the 36th EO was calculated and shown in Figure 27. It can be seen that the 36th EO resonant vibration happens at the rotating speed of 3705 RPM. In this case, the corresponding resonant frequency was calculated as 2223 Hz, which is close to the result obtained by the reconstruction algorithm in Section 3.1.

Finally, resonant frequencies obtained by different methods are compared in Table 2. Taking the theoretical value as a baseline, we can find that absolute estimation errors of the proposed algorithms in Section 3.1 and Section 3.2 are equal to only 4% and 3.86%, respectively. Therefore, the experimental results demonstrate that the proposed algorithms can be feasible for BTT-based on-line vibration monitoring under variable rotating speeds.

Similarly, it should be emphasized that it is impossible to accurately detect the vibration amplitude associated for each order due to well under-sampled BTT signals. Thus, the actual aim of the proposed methods is not to detect the amplitude associated to specific known response frequency.

## 6. Conclusions

Nowadays, BTT is generally regarded as an advanced technique of on-line monitoring vibrations of high-speed rotating blades. In practice, blade damages are always formed during variable operation conditions, so that VRS brings a significant obstacle to existing BTT vibration monitoring methods. As for the problems of variable rotation speeds and under-sampled signals, this paper proposed a reconstructed order analysis-based BTT vibration monitoring method. The paper contributes to the discussion in the following ways: (i) Basic structure of angular sampling-based BTT vibration monitoring under VRS is presented; (ii) A band-pass sampling-based EO reconstruction algorithm is proposed for uniform BTT sensor configuration, so that few BTT sensors can be used to extract high EOs; (iii) A periodically non-uniform sampling-based EO reconstruction algorithm is proposed for non-uniform BTT sensor configuration, so that fewer BTT sensors can also be used to extract high EOs; and (iv) experimental results testify that the proposed algorithms can accurately capture characteristic EOs of synchronous and asynchronous blade vibrations under VRS, so that the demand of classical BTT methods on constant rotating speed is released. Therefore, the proposed methods are promising for accurately detecting blade damages during operations. In future, we will carry out experiments for blade damages and study on extracting damage features by using the method in this paper. In addition, other damage features can be studied, such as circular domain features [31]. At the same time, it will be valuable to compare the results of this paper with conventional strain gauge measurements. It is suggested that this kind of research can be undertaken in future works.

## Figures and Tables

**Table 1:** Mechanical and geometrical properties of the blade used in the experiment.

Parameters	Values
Blade material	40 Cr steel
Blade length	34 mm
Blade width	11 mm
Blade thickness	3 mm
Blade tip radius	103 mm

**Table 2:** Comparison of resonant frequencies obtained by different methods.

Different Methods	Resonant Frequency	Absolute Errors
Theoretical calculation	2140.4 Hz	–
The GARIV method	2270 Hz	6.05%
Algorithm in Section 3.1	2226 Hz	4.00%
Algorithm in Section 3.2	2223 Hz	3.86%
